# Regulatory RNAs in the Less Studied Streptococcal Species: From Nomenclature to Identification

**DOI:** 10.3389/fmicb.2016.01161

**Published:** 2016-07-26

**Authors:** Mohamed A. Zorgani, Roland Quentin, Marie-Frédérique Lartigue

**Affiliations:** ^1^ISP, INRA, Equipe 5 “Bactéries et Risque Materno-foetal", Faculté de Médecine, UMR 1282, Université François Rabelais de Tours, ToursFrance; ^2^Service de Bactériologie Virologie et Hygiène Hospitalière, Centre Hospitalier Régional Universitaire de Tours, ToursFrance

**Keywords:** small RNAs, non-coding RNA, *Streptococcus agalactiae*, *Streptococcus mutans*, *Streptococcus thermophilus*, Regulatory RNA nomenclature

## Abstract

Streptococcal species are Gram-positive bacteria involved in severe and invasive diseases in humans and animals. Although, this group includes different pathogenic species involved in life-threatening infections for humans, it also includes beneficial species, such as *Streptococcus thermophilus*, which is used in yogurt production. In bacteria virulence factors are controlled by various regulatory networks including regulatory RNAs. For clearness and to develop logical thinking, we start this review with a revision of regulatory RNAs nomenclature. Previous reviews are mostly dealing with *Streptococcus pyogenes* and *Streptococcus pneumoniae* regulatory RNAs. We especially focused our analysis on regulatory RNAs in *Streptococcus agalactiae*, *Streptococcus mutans*, *Streptococcus thermophilus* and other less studied *Streptococcus* species. Although, *S. agalactiae* RNome remains largely unknown, sRNAs (small RNAs) are supposed to mediate regulation during environmental adaptation and host infection. In the case of *S. mutans*, sRNAs are suggested to be involved in competence regulation, carbohydrate metabolism, and Toxin–Antitoxin systems. A new category of miRNA-size small RNAs (msRNAs) was also identified for the first time in this species. The analysis of *S. thermophilus* sRNome shows that many sRNAs are associated to the bacterial immune system known as CRISPR-Cas system. Only few of the other different *Streptococcus* species have been the subject of studies pointed toward the characterization of regulatory RNAs. Finally, understanding bacterial sRNome can constitute one step forward to the elaboration of new strategies in therapy such as substitution of antibiotics in the management of *S. agalactiae* neonatal infections, prevention of *S. mutans* dental caries or use of *S. thermophilus* CRISPR-Cas system in genome editing applications.

## Introduction

Gene regulation is fundamental for bacteria to adapt to environmental changes. Therefore, bacteria have developed and evolved strategies to tightly control genetic networks in response to diverse extracellular stimuli (Casadesus and Low, 2006). Bacterial regulatory pathways involving small RNAs (sRNAs) are now being elucidated and their functions are coming to light, particularly in regards to bacterial physiology, adaptation and pathogenesis as well as to plasmid replication control (Cavanagh and Wassarman, 2014; Brantl, 2015; Lopez-Aguilar et al., 2015). Beside transcriptional and translational gene regulation (activation or inhibition) through direct interaction with mRNAs, regulatory RNAs perform a variety of other mechanisms on their targets including: (i) sequestering molecules (e.g., binding to proteins; Babitzke and Romeo, 2007), (ii) affecting the expression of *cis* elements (e.g., riboswitches; Winkler and Breaker, 2005) and (iii) targeting invading cognate DNA [e.g., CRISPR RNAs (clustered regularly interspaced short palindromic repeats RNAs); Garneau et al., 2010]. The importance of sRNAs-mediated regulatory mechanisms depends on a fine-tuning of the expression of an armament of virulence factor genes (Papenfort and Vogel, 2010; Mann et al., 2012). Considering the functional roles of these RNAs, they could be targets for the development of future therapeutic drugs (Lalaouna et al., 2014).

Streptococcal species can colonize and invade humans and animals. They mostly exhibit an asymptomatic interaction within their hosts. However, several of them are well-known pathogenic species responsible for severe and live-threatening infections in humans. Infections can be exclusive to human, as it is the case for *Streptococcus pyogenes* leading to a substantial mortality and morbidity (Carapetis et al., 2005). They can also switch from a benign and commensal stage to an infectious one, like for *Streptococcus pneumoniae* (Gamez and Hammerschmidt, 2011) and the neonatal opportunistic pathogen *Streptococcus agalactiae* (Maisey et al., 2008; Krzysciak et al., 2013). In the last decades, the study of these pathogens has increased intensively due to the spreading of antibiotic resistance. Therefore, researchers are continuously invited to look for novel targets and to develop innovative therapies to combat streptococcal infections. With regard to the current knowledge on regulatory RNAs functions and modes of action, sRNAs are expected to play central roles in the bacterial regulatory networks, physiology, adaptation and virulence. Thus, they are thought-out as alternative types of regulators for adaptive gene regulation and drugs design.

Previous reviews on streptococcal sRNAs were mainly restricted to two streptococcal species: *S. pyogenes* and *S. pneumoniae* (Le Rhun and Charpentier, 2012; Patenge et al., 2013; Brantl and Bruckner, 2014; Miller et al., 2014; Wilton et al., 2015; Bouloc and Repoila, 2016). However, the *Streptococcus* genus includes a large group of commensal and pathogenic Gram-positive bacteria. This review aims to provide more complete and comprehensive state of the art on the hidden part of streptococcal regulatory RNAs. It focuses on *S. agalactiae*, *Streptococcus mutans*, *Streptococcus thermophilus* sRNAs, as well as other less studied species, and brings in light their sRNome with reference to recent advances in the fast moving area of bacterial sRNAs. The clearness of scientific reports is intimately linked to the simplicity of the nomenclature been used. Thus, we dedicated the first chapter of this review to present and reemphasize the use of bacterial regulatory RNAs nomenclature. Please note that in order to put forward *Streptococci* sRNome, we will not discuss small RNAs from other Gram-positive bacteria (Romby and Charpentier, 2009; Brantl and Bruckner, 2014).

## Bacterial Regulatory RNAs: Terminology Issues

Regulatory RNAs are commonly divided into three major groups: the first group includes *cis-*acting elements located on untranslated regions (UTRs) of a translated mRNA. They control the expression of their enclosed gene(s) through the modulation of their secondary structures or stability (Johansson et al., 2002). A second class of sRNAs are antisense RNAs (asRNAs), whose regulatory activity occurs through perfect base-pairing to the mRNA encoded by the opposite DNA strand molecule (Thomason and Storz, 2010). The last group of sRNAs, also known as *trans*-acting sRNAs, includes RNAs that target other RNAs, foreign DNA, or proteins (Gottesman and Storz, 2010). In the case of RNA targets, this group of sRNAs often shares a limited complementarity with their targets; thus, they can bind to multiple mRNAs. This type of interaction requires, often for stabilization issues, the RNA chaperone protein Hfq, which is absent in the *Streptococcus* genus and other Gram-positive bacteria (Brennan and Link, 2007; Nielsen et al., 2009).

As it is the case in proteomics and genomics (Desvaux et al., 2009), regulatory RNAs nomenclature issues raise some confusion in the field of bacterial regulatory networks. For instance, the use of the descriptors “*cis*-acting” or “*cis*-encoded” sRNA to describe an “antisense sRNA,” or the confusion between the use of “*trans*-encoded” and/or “*trans*-acting” sRNA, or even the use of different nomenclatures “Small *trans*-encoded RNA” (Nielsen et al., 2009), “Small regulatory RNA” (Frohlich and Vogel, 2009), “*trans*-encoded sRNA” (Ross et al., 2013), and “Small non-coding RNAs” (Xia et al., 2012) to cite a regulatory RNA. Thus, it is useful to reemphasize, and to remind the reader on regulatory RNA nomenclature.

**FIGURE 1:**
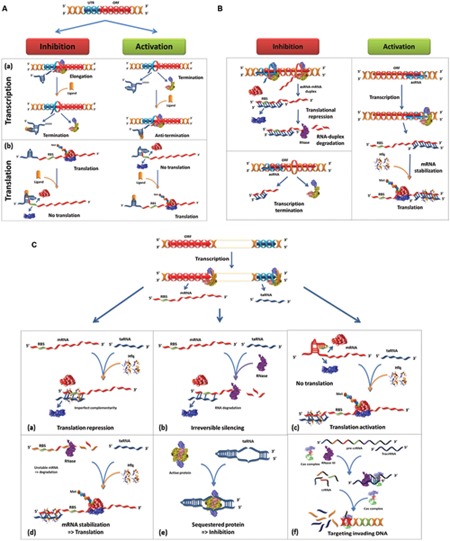
**(A)** c*is*-acting sRNAs (caRNAs) and their mechanism of action. **(a)** 5′ UTR-mediated transcriptional regulation. (Left) In the absence of ligand, transcription is initiated through a permissive stem-loop region, an aptamer region in the 5′ UTR (blue) of an open reading frame (ORF, red). In the presence of ligand, the aptamer region assumes a conformational change and is stabilized by the direct recognition of ligand. The complex (5′ UTR/Ligand) leads to the formation of a U-rich hairpin that acts as a transcriptional terminator. (Right) In the absence of ligand, the transcription is initially attenuated. The recognition of the ligand by the U-rich hairpin leads to the disruption of the stem-loop and thus to the transcription anti-termination or activation. **(b)** 5′ UTR-mediated translational regulation. (Left) In the absence of ligand, the aptamer region forms an anti-terminator hairpin. The ribosome binding site (RBS, green) is available, and mRNA translation is initiated. Upon ligand binding, the access to the RBS is hindered, preventing translation. (Right) In the absence of ligand, translation is inhibited due to a sequestered RBS. The recognition of the ligand by the stem-loop releases the RBS and translation can be initiated. In other cases, the ligand can be replaced by a temperature-dependent mechanism, where the mRNA adopts a secondary structure that hinders the access to the RBS (*Listeria monocytogenes*). **(B)** Mechanisms of mRNA regulation by antisense RNAs (asRNA). (Left upper) The binding of asRNA (blue) to the TIR (translation initiation region) leads to translation repression. The RNA duplex (asRNA-sense RNA) can be degraded by RNase III, a double strand specific endoribonuclease conserved in all the three kingdoms (Cho and Kim, 2015); or by RNase E which belong to the Gram-negative bacteria degradosome (Stazic et al., 2011). (Left lower) The asRNA can bind to the 5′ end of the target mRNA and causes changes in the target RNA structure leading to transcription termination. (Right) The complex asRNA-Hfq can also stabilize target mRNA for translation activation. **(C)** *trans*-encoded sRNAs (treRNAs)-based regulatory mechanisms of mRNA expression. treRNAs act through an imperfect basepairing to mRNA targets. The treRNA/mRNA duplex is stabilized by Hfq and can either repress **(a–b)**, **(c)** activate or **(d)** stabilize mRNA translation. **(e)** It was also demonstrated that treRNAs can bind to proteins to inhibit and/or modify their activity. **(f)** tracrRNAs are a treRNA involved in the maturation of crRNAs through the interaction with the complex CRISPR-Cas9, pre-crRNA and RNase III. The processed crRNAs target the invading DNA.

In this review section, we propose a simplified and coherent nomenclature, which we believe necessary to develop comprehensible and logical thinking when describing bacterial regulatory RNAs. We propose first to revise the division of regulatory RNAs into two major categories (**Table 1**). The first category are the *cis*-acting RNAs. The second one, are the *trans*-acting sRNAs. This category comprises the *cis*-encoded sRNAs, known as antisense RNAs, and the ***t****rans*-**e**ncoded sRNAs, which we refer to them in this review as “treRNAs.” We propose also the use of other abbreviations for some sRNAs categories. For example, we advise the use of “caRNA” and “traRNA” rather than “***c****is*-**a**cting sRNA” and “***t****rans*-**a**cting sRNA,” respectively. Although, few examples are listed for each category of traRNAs (**Table 1**), the proposed list must be updated continuously since our knowledge on bacterial RNA keep changing. An exception is for the descriptor “ncRNAs” generally used to describe regulatory RNAs that do not encode for proteins, known as “non-coding RNAs,” and which can be applied to all regulatory RNA categories. As shown in **Figures 1A–C** regulatory RNAs nomenclature depends on their function and mode of action. The caRNAs can act on transcriptional or translational levels (**Figure 1A**), asRNAs promote transcription attenuation or activation/inhibition of translation (**Figure 1B**). The treRNAs can repress (**Figures 1Ca–b**), or activate translation (**Figures 1Cc–d**), sequester proteins (**Figure 1Ce**) or participate in degrading invading DNA (**Figure 1Cf**).

**Table 1:** Bacterial regulatory RNAs nomenclature and mode of action.

Bacterial regulatory RNAs			
**Mode of action Nomenclature**		**Abbreviations**	**Description**

***cis*-acting RNAs**		caRNA	The descriptor “*cis*-acting RNA” is used to describe regulatory RNAs that act (Ruiz de los Mozos et al., 2013), or are located at the untranslated regions (UTRs) of mRNA molecules (Gossringer and Hartmann, 2012).

	**U**n**T**ranslated**R**egion	UTR	UTRs are *cis*-acting RNAs located on untranslated regions of a coding transcript. They can activate/inhibit translation by releasing/hindering the RBS, respectively. They can also participate in the mRNA transcript stabilization (Gossringer and Hartmann, 2012). The base pairing between 5′- and 3′-UTRs can also control mRNA translation (Ruiz de los Mozos et al., 2013).
	
	**5**′ **U**n**T**ranslated**R**egion	5′ UTR	5′ UTRs are RNAs that act on the 5′-end of the mRNA transcript. They modulate the expression of the downstream genes by controlling the access of the ribosome to the translation initiation region (TIR). This class of UTRs includes Riboswitches, non-transcribed RNA, and independent transcripts. They play an important role in bacterial metabolism control. The modulation of the expression of the 5′ UTR-downstream genes occurs through a metabolite-induced alteration of the RNA secondary structure, such as tRNAs (Loh et al., 2009).
	
	**3**′ **U**n**T**ranslated**R**egion	3′ UTR	3′ UTRs are RNAs that act on the 3′-end of the mRNA transcript. They constitute the region of the mRNA that immediately follows the translation termination codon (TTC; Chao et al., 2012).

***trans*-acting RNAs (traRNAs)**			

	***cis*-encoded** (**a**nti**s**ense)**RNA**	asRNA	asRNAs are *cis*-encoded RNAs that act on the mRNA molecule encoded by the opposite DNA strand. They present a perfect base-pairing with their mRNA target (Thomason and Storz, 2010). We also distinguish toxin–antitoxin systems (Lee and Hong, 2011) and asRNAs encoded by the streptococcal pIP501 plasmid (Brantl and Wagner, 1996), or long asRNAs described in eukaryotes (Vadaie and Morris, 2013).
	
	**5**′ **a**nti**s**ense RNA	5′ asRNA	5′ asRNAs are asRNAs that act on the 5′-end of the sense mRNA target. They activate or inhibit the translation of the mRNA transcript (Thomason and Storz, 2010). 5′ asRNAs can also act by transcription attenuation, which is frequently found on plasmids (Thomason and Storz, 2010).
	
	**3**′ **a**nti**s**ense RNA	3′ asRNA	3′ asRNAs are asRNAs that act on the 3′-end of the sense mRNA target. The role of the 3′ asRNAs is not yet well-deciphered. However, it was suggested that they participate in the transcript stabilization (Opdyke et al., 2004; Padalon-Brauch et al., 2008).
	
	***trans*-encoded RNA**	treRNA	treRNAs are RNAs that target distant and/or different RNAs, eliminate invading cognate DNA, or form ribonucleo-protein complexes (Storz et al., 2011). We can also distinguish in this class: housekeeping RNAs such as 6S RNA, RNase P RNA (Brantl, 2009), overlapping ncRNA (Kumar et al., 2010), and operon RNAs (Kreikemeyer et al., 2001).
	
	***c****ia*-dependent **s**mall RNA	csRNA	csRNAs are treRNAs that are transcribed from the promoters associated to CiaRH two-component system. They present a complementarity to the Shine-Dalgarno sequence and the translation initiation codon (AUG; Marx et al., 2010).
	
	**m**icroRNA-size sRNA	msRNA	msRNAs are a very small RNAs (15 to 26 nts) that are first described in *Streptococcus mutans*. The role of these microRNA-like sRNAs in bacterial gene regulation is not yet understood (Lee and Hong, 2011; Kang et al., 2013).
	
	**CR**ISPR **R**NA	crRNA	As a part of the CRISPR (clustered regularly interspaced short palindromic repeat) system, the crRNAs act as treRNAs targeting and processing foreign DNA (Karvelis et al., 2013).
	
	***tr****ans*-**a**ctivating **CR**ISPR RNA	tracrRNA	In the CRISPR/Cas system, tracrRNAs are in charge of the activation of the pre-crRNAs (Karvelis et al., 2013).

## *Streptococcus agalactiae*: What Is Behind the Hidden sRNome?

Apart from its commensal colonization of the gastrointestinal and urogenital tracts, *S. agalactiae*, known also as Group B *Streptococcus* (GBS), has become a predominant cause of invasive infections in neonates and causes pneumonia, septicemia, and meningitis (Maisey et al., 2008; Krzysciak et al., 2013). However, the current knowledge on the sRNome encoded by the GBS chromosome remains limited, even though the sRNAs can play an important role in virulence of various Gram-positive bacteria including *Streptococcus* (Patenge et al., 2013). Two *in silico* studies were conducted on the sequenced genome of *S. agalactiae* NEM316. The first study aimed to predict sRNAs associated to the CiaRH two component system; we refer to them as csRNAs (Marx et al., 2010; **Table 1**). The CiaRH response regulator in *S. agalactiae* plays an important role in promoting the innate immune system, and intracellular survival (Quach et al., 2009). The search for CiaR-activated promoter (NTTAAG-N5-TTTAAG) and the transcriptional terminator sequences within the intergenic regions led to the identification of four putative csRNAs (csRNA10, csRNA11, csRNA12, and csRNA13; Marx et al., 2010). However, no experimental evidence on the expression of *S. agalactiae* csRNAs was reported in this study. The presence of complementarity to translation initiation regions in all the csRNAs suggests that they can bind to mRNAs and block translation initiation by hindering the access of ribosomes. Of note, the expression of csRNA10, csRNA11, and csRNA12 was recently confirmed and their up-regulation by acidic conditions was demonstrated using northern blot analysis in *S. agalactiae* NEM316 strain (Rosinski-Chupin et al., 2015). The control by the CiaRH two-component system of these csRNAs underlines their potential role in *S. agalactiae* acidic stress adaptation in the urogenital tract.

The second *in silico* study was based on prediction of Rho-independent terminators (RITs) as a specific signature of sRNA gene loci. One hundred ninety-seven sRNAs were detected on the genome of *S. agalactiae* NEM316 (Pichon et al., 2011). The expression of 10 out of the 197 sRNAs was confirmed by RT-PCR and northern blot hybridization. One of the sRNAs candidates (SQ517) detected in this study was previously reported as putative csRNA (csRNA12; Marx et al., 2010). The over-expression of SQ18 asRNA significantly decreased, more than 100-fold, the level of its target *gbs0031* mRNA, which encodes a surface immunogenic protein (Sip). A translational fusion system showed also that the over-expression of SQ18 was associated with fourfold decrease in the amount of *gbs0031::gfp* mRNA, suggesting that SQ18 acts as a negative post-transcriptional regulator.

A more recent study led to the identification of 125 sRNAs: 39 caRNAs, 39 asRNAs, and 47 treRNAs (Rosinski-Chupin et al., 2015). Ten of these sRNAs were differentially expressed in response to acidic stress. The expression of Srn015 (csRNA10), Srn024 (csRNA11), Srn070 (csRNA12), Srn085 (csRNA13), Srn071 and Srn082 was induced by more than two-fold at pH of 5.2 (natural pH of the genitourinary tract). In contrast, Srn046, Srn056, Srn057, and Srn073 were down regulated in the same experimental condition. All csRNAs identified in this study were previously predicted (Marx et al., 2010), indicating that sRNA *in silico* prediction is a powerful tool for csRNA identification in *Streptococci*.

Other sRNAs were described previously in mobile genetic elements of *S. agalactiae*, like the asRNAs involved in the replication control of the pIP501 and pMV158 plasmids (Brantl and Behnke, 1992; del Solar and Espinosa, 1992; Brantl and Wagner, 1994, 1996; Brantl, 2015; Lopez-Aguilar et al., 2015). Both pIP501 and pMV158 were originally isolated from clinical strains of *S. agalactiae* and exhibit a broad range of hosts. The asRNAs RNAIII and RNAII encoded by pIP501 and pMV158, respectively, have been found important for the inhibition of expression of the plasmids essential genes. While pIP501 RNAIII asRNA exhibits unusual stability, the RNAII encoded by pMV158 did not require the formation of a kissing complex for efficient binding to its mRNA target or for inhibition of gene expression. Due to a limited number of available genome sequences, the *S. agalactiae* sRNome remains largely unknown.

## *Streptococcus mutans* sRNAs: From Very Small to a Toxic Mood

Proposed to be the new Gram-positive paradigm, *S. mutans* is a host-associated dental pathogen (Lemos et al., 2013). This bacterium is considered to be a cariogenic organism responsible for the formation of dental biofilm matrix and some occasional infective endocarditis. Beside its ability to survive at low pH and to produce large quantities of organic acid, *S. mutans* presents an ease of genetic manipulation. This fluency is due to the presence of a two competence regulatory system ComR/ComS, which makes the bacterium transformable in different culture conditions (Son et al., 2012). In *S. mutans*, sRNAs were first described after high-throughput experiments and a kingdom-wide prediction of bacterial sRNA-encoding genes using SIPHT (sRNA identification protocol using high-throughput technologies) method (Livny et al., 2008). This strategy identifies new treRNAs candidates by co-localizing their intergenic regions and the Rho-independent terminators. Eighteen treRNA genes of *S. mutans* were predicted, however, their functions as those of most *S. mutans* treRNAs remain elusive (Livny et al., 2008). Biocomputational studies predicted that three treRNAs were putatively regulated by CiaRH (csRNA23-1, csRNA23-2, and csRNA24; Marx et al., 2010). As mentioned earlier, csRNAs should get deeper attention owing to their potential role in *S. mutans* virulence through the interaction with the CiaRH regulon.

A new member of the treRNAs category, baptized “miRNA-size small RNAs” or “msRNAs” was recently described in *S. mutans* (Lee and Hong, 2011; **Table 1**). In eukaryotes, microRNAs (miRNAs) are 19–23 nucleotides (nts) non-coding RNAs that act as post-transcriptional regulators leading mainly to gene silencing (Bartel, 2004). However, so far no publication reports sRNAs with comparable size to miRNAs in bacteria. A deep sequencing approach suggested that detected reads corresponding to 15 and 26 nts were putative msRNAs (Lee and Hong, 2011). These RNAs may result from processed RNAs. In addition, northern profiles show discrete band of msRNA-428 suggesting that this msRNA is not produced through a random RNA degradation. Likewise, the expression of seven msRNAs was validated by qRT-PCR. Currently, the role of msRNAs in gene regulation remains unclear.

The ability of *S. mutans* to metabolize a multitude of carbohydrate sources and to colonize the tooth surface is intimately linked to its ability to survive at low pH. Xia et al. (2012) described for the first time in *S. mutans* a pH-dependent sRNA. This treRNA, L10-leader sRNA, was predicted by combining different bioinformatics approaches. A total number of 334 sRNAs were predicted, however, only the L10-leader was verified by qRT-PCR and northern blot analyses due to its high abundance. The expression level of the L10-leader was growth phase-dependent, and highly affected by pH. In clinical strains, its expression was higher in the most adhering and acidic strains and may be linked to virulence (Xia et al., 2012).

Experimental identification or bioinformatics screens alone are not enough to detect all encoded sRNAs, since their expression often depends on environmental conditions (growth phase, stress condition…). Combining *in vivo* and *in silico* approaches is the best way to identify a large number of bacterial sRNAs. Rho-independent terminator-based, transcription initiation signal-based and RNAs prediction methods were coupled to RNA-seq approach to identify the sRNome of *S. mutans* UA159, under carbohydrate repression as well as in the presence/absence of the catabolite control protein CcpA (Zeng et al., 2013). Among the 114 sRNAs expressed, five were differentially expressed in *S. mutans* UA159 grown in glucose versus galactose, and two treRNAs were deregulated in the *ccpA* mutant. The fact that these treRNAs are expressed in carbohydrate- and/or *ccpA*-dependent manner highlights that they may be involved in the regulation of the *S. mutans* carbohydrate metabolism.

Bacteria have also developed many adaptive systems including Toxin–Antitoxin (TA) modules. These small genetic elements express a stable toxic protein (toxin), and a proximate antitoxin. The nature of the antitoxin, RNA or protein, defines the type of the TA module (Wen and Fozo, 2014). In the type I TA system, the antitoxin is an RNA molecule that controls the mRNA stability and translation of the corresponding toxin molecule. It is proposed that such systems help bacteria to survive to environmental stress, and when exposed to high concentrations of antibiotics through the formation of persister cells (Gerdes et al., 2005). Recently, a functional type I TA system was described for the first time in *S. mutans* UA159 (Koyanagi and Levesque, 2013). This TA module is located in a small intergenic region of 318 nts. The toxin (Fst-Sm) is a small hydrophobic peptide and the antitoxin (srSm) is an asRNA of approximately 70 nts in length. The 5′ end mapping of Fst-Sm and srSm transcripts indicates that they are both transcribed through a direct tandem repeat. It was also demonstrated that the expression of srSm represses the expression of the toxin when fused to a GFP gene reporter. Overexpression of the toxin Fst-Sm induced *E. coli* cell death, and was lethal in *S. mutans*. Ectopic expression of the TA module in *S. mutans* was followed with a decrease in persistent cell formation. Altogether, these results suggest that the *S. mutans* TA systems are implicated in infections caused by oral bacteria. *S. mutans* has the ability to cope with complex environments such as oral cavities, being responsible for dental caries and chronic infections. Deciphering the role of sRNAs in *S. mutans* regulatory networks may help providing some clues to design novel therapeutic strategies for dental caries.

## *Streptococcus thermophilus* sRNome: Too Rich or Too Poor?

*Streptococcus thermophilus* is a lactic acid bacterium used in yogurt manufacturing and also commonly found in fermented milk products. *S. thermophilus* is considered as non-pathogenic for food industries (Blomqvist et al., 2006). In genetics, the name of *S. thermophilus* is intimately linked to the bacterial immune system known as CRISPR-Cas system (Deveau et al., 2008; Sapranauskas et al., 2011; Karvelis et al., 2013; Carte et al., 2014). This sRNA-based immune system protects bacteria from invading DNA, such as viruses and plasmids (**Table 1**). An important player in this process, as it is the case for the Cas9 protein and the crRNA (CRISPR RNA), is the tracrRNA (*trans*-activating CRISPR RNA). Northern blot profiling revealed the presence of a tracrRNA located upstream of the *cas9* gene of the CRISPR3-Cas in *S. thermophilus* LMD-9 genome (Karvelis et al., 2013). This tracrRNA is involved in crRNA biogenesis through the pre-crRNA maturation, and is required for the Cas9-mediated interference. In another study, it was demonstrated that this treRNA, with the help of the RNase III, can stimulate the cleavage within the CRISPR repeats generating the crRNA units (Carte et al., 2014).

Other sRNAs were reported for *S. thermophilus*. Five csRNAs were predicted on *S. thermophilus* CNRZ1066 genome and one csRNA on the plasmid pSt0 (Marx et al., 2010). The expression of the latter csRNA was verified by northern blot. The *S. thermophilus* CiaRH system is inactivated by the presence of a stop codon interrupting the *ciaR* gene, and a frame shift mutation in *ciaH*. This could be a result of a particular adaptation to the dairy niche leading to loss of the function of virulence genes. Another four treRNAs were predicted by homology comparison to *S. pyogenes* MGAS315 sRNome (Tesorero et al., 2013). The expression of these treRNAs needs to be verified.

## *Streptococci* sRNAs: A Large Species Panel

Currently, 67 different *Streptococcus* species are reported on the NCBI database and their genome sequenced and annotated. Nevertheless, only a few of them have been the subject of studies pointed toward the characterization of sRNAs. Bioinformatics approaches have been widely used to detect sRNAs in bacterial genomes. Tesorero et al. (2013) reported that among the 67 available genomes, 23 different *Streptococcus* genomes harbor at least one sRNA homolog to the sRNAs detected for *S. pyogenes* MGAS315 strain. Moreover, species as *Streptococcus suis, Streptococcus uberis, Streptococcus mitis, Streptococcus oralis, Streptococcus sanguinis, Streptococcus dysgalactiae* were also reported to harbor more than two csRNAs (Marx et al., 2010).

For *S. suis*, a pig pathogen, a differential RNA-sequencing approach was conducted on the strain P1/7 grown in rich medium and biological fluids (pig blood and cerebrospinal fluid) and allowed the identification of 29 sRNAs (Wu et al., 2014). Ten of them were predicted to be conserved in 34 pathogenic *Streptococcus* species. The deletion of five treRNAs led to the attenuation of *S. suis* virulence in a zebrafish infection model. *S. suis* mutant strains were more sensitive to killing by pig blood following the deletion of three other treRNAs as compared with the wild-type. This study shows also that genes involved in the synthesis of capsular polysaccharide were differentially regulated in blood and cerebrospinal fluid. The role of *S. suis* sRNAs in virulence, the mechanism of interaction and regulation with their mRNA targets need to be investigated.

## Future Prospects and Concluding Remarks

The aim of this focused review was to bring in light the sRNomes of *Streptococcus* species. As cited earlier, understanding *S. agalactiae* sRNome can constitute one step forward in providing new strategies for the substitution of antibiotics in the treatment of neonatal infections. A major cause of dental caries, *S. mutans*, harbors a significant number of sRNAs that can play an important role in its virulence. *S. thermophilus* is the only *Streptococcus* species used in food industry. The use of CRISPR-Cas system in genome editing through genetic engineering is a very promising tool in bacterial genome new function design. The future challenges in the study of sRNAs in bacteria are the identification of their targets and the understanding of their regulatory mechanism. In *Streptococci* cited in this review, there is a lack between the identification of sRNAs and their functional characterization. Future works should be done on the mechanistic level; target identification and mechanism of regulation, in order to better appreciate these regulatory networks.

## Author Contributions

MZ wrote the manuscript. M-FL and RQ participated in manuscript elaboration and correction.

## Conflict of Interest Statement

The authors declare that the research was conducted in the absence of any commercial or financial relationships that could be construed as a potential conflict of interest.
